# The Current Status of Virtual Autopsy Using Combined Imaging Modalities: A Scoping Review

**DOI:** 10.3390/jcm14030782

**Published:** 2025-01-25

**Authors:** Romica Cergan, Iulian Alexandru Taciuc, Mihai Dumitru, Daniela Vrinceanu, Felicia Manole, Nicoleta Sanda, Andreea Nicoleta Marinescu

**Affiliations:** 1 Anatomy Department, Carol Davila University of Medicine and Pharmacy, 050474 Bucharest, Romania; r.cergan@gmail.com; 2 Pathology Department, Carol Davila University of Medicine and Pharmacy, 050474 Bucharest, Romania; alexandertaciuc@gmail.com; 3 ENT Department, Carol Davila University of Medicine and Pharmacy, 050474 Bucharest, Romania; vrinceanudana@yahoo.com; 4 Department of ENT, Faculty of Medicine, University of Oradea, 410087 Oradea, Romania; manole.felicia@gmail.com; 5 General Surgery Department, Carol Davila University of Medicine and Pharmacy, 050474 Bucharest, Romania; nico.sanda@yahoo.com; 6 Imaging Department, Carol Davila University of Medicine and Pharmacy, 050474 Bucharest, Romania; andreea_marinescu2003@yahoo.com

**Keywords:** virtopsy, postmortem imaging, alternative non-clinical autopsy

## Abstract

**Background/Objectives**: Virtual autopsy (virtopsy) is a new domain of research for interdisciplinary teams of radiologists and forensic specialists. This scoping review aims to underline the current state-of-the-art research using combined imaging modalities. **Methods**: We searched the PubMed database using the term virtopsy for articles that are available in free full text, indexed in the Medline Database, and published in English. The query returned 49 articles on this subject that have been published since 2002. **Results**: The main imaging modalities used for postmortem imaging were computed tomography (PMCT), angiography (PMCTA), magnetic resonance imaging (PMMRI), and ultrasonography (PMUS). PMCT is highly effective for detecting complex osseous injuries, tracing bullet trajectories, or identifying characteristic findings in drowning cases. PMCTA is valuable for evaluating vascular lesions, particularly in natural death cases. PMMRI is superior in analyzing soft tissues, including brain and spinal structures, cerebrospinal fluid, microbleeds, and laryngohyoid lesions, and identifying cardiomyopathies in young individuals. PMUS serves as an alternative, and its portability also allows for use in forensic settings. One specific situation observed was the increased number of studies published about virtopsy during the COVID-19 pandemic. Another aspect is the increased focus on this alternative to conventional autopsy in the regions where maneuvering of the deceased is limited according to cultural and social customs. **Conclusions**: We underline the advantages and limitations of each imaging modality used for virtopsy. Further studies need to be developed in order to gather supplementary data regarding the use of these imaging modalities in the new era of artificial intelligence in medicine.

## 1. Introduction

The traditional method of postmortem investigation involves opening all of a body’s cavities. To complement or even replace this approach, techniques such as body fluid aspiration and various tissue biopsies have been introduced [1]. Over time, several challenges and limitations have been identified in conventional autopsies. As previously noted, extensive incisions result in bodily mutilation and disfigurement. Additionally, there is the potential for subjective variation and interpretation errors between different examiners [2]. Following the procedure, the body is returned to the family for funeral arrangements, making it difficult to conduct a second autopsy to verify initial findings, thereby limiting the reproducibility of conventional autopsies [3]. Furthermore, certain religious and cultural beliefs prohibit traditional autopsies, which can impede the pursuit of justice [4]. Besides these limitations, other concerns were raised regarding the personnel’s exposure to bodily contaminants of the corpse and transmission of infection [5].

Fundamentally, virtopsy is a technologically advanced method for conducting autopsies, characterized by its minimal invasiveness and wide range of applications, particularly in forensic science, medical education, and mass disaster management. The term is derived from the combination of “virtual” and “autopsy” and was trademarked in 2011 by Richard Dirnhofer, former head of the Institute of Forensic Medicine at the University of Bern, Switzerland [6,7].

The goal of virtual autopsy is to minimize the subjectivity inherent in traditional autopsies, where findings are primarily based on the examiner’s visual assessment. In contrast, virtual autopsy relies heavily on radiological imaging technologies, with the doctor’s role focused mainly on interpreting the results rather than direct visualization [3].

The advantages of virtopsy are considerable. The literature highlights the technique’s superiority in comprehensive whole-body analysis, offering excellent evaluation of skeletal pathologies and improved visualization of areas such as the face, hands, and legs, which are often not thoroughly examined in traditional autopsies. Additionally, virtopsy provides enhanced detection of air inclusion lesions [8]. The use of 3D reconstructions can both identify and support evidence in legal proceedings, with the added benefit that the data can be stored and reviewed an unlimited number of times [5]. Although the cardiovascular system may appear inaccessible with native CT or MRI, postmortem angiography remains a viable option and provides excellent evaluation in cases involving hemorrhages [8].

Virtopsy has certain limitations, as outlined in the literature. First, it requires specially trained personnel, and the cost of the equipment and instruments is significant [9]. Additionally, due to its “virtual” nature, virtopsy is unable to provide information on histopathological features, DNA, bacterial presence, chemical substances, or toxins within the deceased body [10]. While virtopsy offers valuable information in legal cases where cultural or religious beliefs prohibit standard autopsy, it is important to note that digital images can be altered, such as by changing, moving, or deleting pixels. Therefore, strict protocols for image processing are essential to ensure the integrity and authenticity of the visual data [9].

This paper aims to compile and summarize existing research on this topic. While numerous academic studies have been published on virtopsy, questions remain regarding its accuracy compared to traditional autopsy, the types of pathologies that are better diagnosed by one method over the other, and whether virtopsy should be used as a preliminary tool to determine the need for a conventional autopsy, or vice versa. Our objective is to evaluate the diagnostic accuracy of a virtopsy in determining the cause of death and to identify the cases in which a virtopsy should serve as a preliminary investigation before conducting a traditional autopsy. We include studies and case reports comparing virtopsy results with traditional autopsies, assessing whether the latter provided additional information. Additionally, we will analyze the other relevant literature reviews that evaluate these findings. The primary outcomes of interest will be the diagnostic accuracy of virtopsy and the extent of information it provides in comparison to conventional autopsy. These aspects are crucial to determine due to their practical implications, such as improving the identification of the cause of death and optimizing management in high-demand departments. Additionally, they hold significant ethical importance, particularly in cases where cultural or religious beliefs prohibit traditional autopsy procedures.

## 2. Materials and Methods

**Figure 1:**
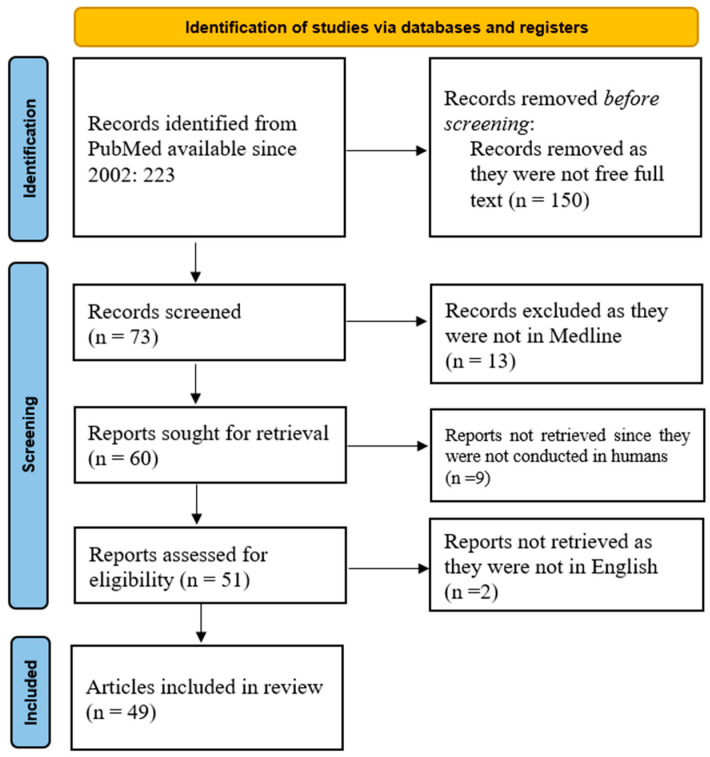
PRISMA flowchart of the current scoping review on virtopsy.

This review followed the Preferred Reporting Items for Systematic Reviews and Meta-Analyses (PRISMA) guidelines to evaluate the diagnostic abilities of virtopsy in forensic practice compared to traditional autopsy in order to determine whether virtopsy should serve as a preliminary investigation before conducting a traditional autopsy. We queried the PubMed database using the keyword “virtopsy”. We obtained 223 manuscripts from the last 22 years to October 2024. The restriction of research to articles where the free full text was available limits the number of usable manuscripts, as shown in Figure 1.

PubMed search results outlined in Figure 1 were imported into one online cloud database. Afterward, two groups of two reviewers each (I.A.T. and M.D.; D.V. and A.M.) independently screened the full texts for inclusion. Differences between reviewers were resolved by another set of two reviewers (R.C. and N.S.). F.M., a co-author from outside our university, was responsible for limiting possible bias.

In order to correctly analyze the results, we divided the results based on the imaging modality used in the studies and, furthermore, the pathologies in which the performance was assessed.

## 3. Results

**Figure 2:**
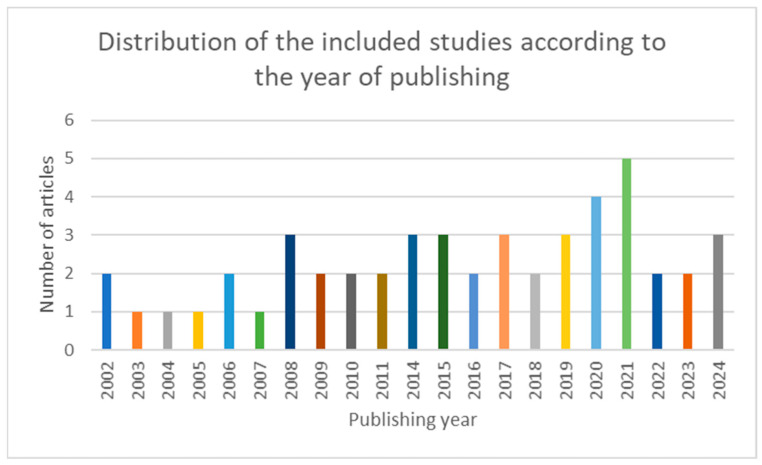
Chart depicting the distribution of the included studies according to the year of publishing.

As presented previously, virtopsy includes more imaging techniques. Interestingly, many published articles were recorded in the years 2020 and 2021 during the COVID-19 pandemic, which is proof of the increased use of noninvasive autopsy in the context of the SARSCOV-2 pandemic, Figure 2.

### 3.1. Postmortem Computed Tomography (PMCT)

Postmortem gas formation, hypostasis, tissue density changes, loss of vascular integrity, and fluid redistribution can progressively alter the original structural characteristics visible on CT images, potentially leading to the misidentification of injuries or diseases, resulting in false positives or negatives [11].

A case report used both autopsy and PMCT in an elevator-related incident. Although the PMCT revealed complex spinal fractures, the conventional autopsy determined that the cause of death was due to severe polytrauma. The multidisciplinary aspect only provided a better reconstruction of the accident, revealing some complex spinal fractures (Jefferson fracture and complete Chance fracture) and spinal deviation proximal to the fracture site [12]. The imaging technique also established superiority in facial and osseous injuries [13], with the three-dimensional reconstruction being able to characterize bone fractures better and provide leads for the subsequent autopsy investigation [14]. Even in cases of high-energy blunt trauma, PMCT demonstrated greater sensitivity in detecting skeletal injuries. However, traditional autopsy remained more effective for identifying thoracic and abdominal visceral injuries [15]. In the case of rib fractures, curved planar reformation may increase rib fracture detection rates of non-radiologists, the technique also provides better overview visualization of the lesions [16]. Unenhanced postmortem CT (PMCT) is not recommended as a substitute for conventional autopsy in cases of fatal traumatic abdominal injuries due to its low sensitivity and negative predictive value [17].

In cases of gunshot wound-related deaths, PMCT has been successfully utilized to detect entry and exit wounds, trace the bullet trajectory, and identify organ damage. However, it provides limited information regarding the extent of lesion invasiveness across different tissue types. Additionally, PMCT does not consistently differentiate between entry and exit wounds, which are critical details in forensic investigations [18,19]. Despite its limitations, PMCT can, in certain cases, provide valuable insights into the sequence of events, helping to resolve controversies surrounding the circumstances that led to the individual’s death [20].

Gas accumulation in intervertebral disks and Simon’s bleeding are characteristic signs observed in hanging deaths. Studies have shown that gas accumulation in the intervertebral disks is more commonly found in younger decedents, while Simon’s bleeding is more likely to be present in cases of complete hanging [21].

In cases of drowning, PMCT can identify characteristic findings, including fluid accumulation in the airways and sinuses, ground-glass opacities, pulmonary overinflation, and retention of foam-like material in the airways (although this is not always visible on imaging). Additional observations may include hyperdense sediments, such as mud or sand, pleural effusions, or fluid in the stomach. However, these imaging features should be interpreted in conjunction with cadaveric signs, diatom analysis, and histopathological examination for a comprehensive diagnosis [22].

A systematic review has previously examined the multidisciplinary approach to laryngohyoid lesions, revealing statistically significant findings. The review concluded that combining PMCT with traditional autopsy detected a higher number of osseocartilaginous injuries than either method alone, with PMCT proving especially effective. These conclusions were drawn from an analysis of twelve studies, underscoring the complementary value of PMCT and autopsy in forensic investigations of laryngohyoid trauma [23]. No correlation was found between epiglottis calcification and an increased incidence of failed endotracheal intubation on PMCT [24]. Consequently, PMCT does not appear to provide essential diagnostic value in assessing such cases.

Regarding brain imaging, in softened and liquified brains, the PMCT’s main strength was the evaluation of intracranial gas accumulations. Apart from partial recognition of ventricular system configuration in some cases, this method is not useful to recognize intracranial structures [25]. There is a case report of a male with a history of cannabis abuse who was found dead after smoking hyoscine butylbromide. Due to the particularity of the case, a comprehensive approach was opted for, and the PMCT found significant cerebral edema and lung edema that were also confirmed through histopathological examination [26]. Beyond diagnostic purposes, postmortem changes in the brain were also analyzed. The results showed that CT brain slices provided a range of quantitative descriptors that could effectively monitor postmortem changes over time, with measurements taken within 0-4 days postmortem [27]. Although the study did not recommend using this technique alone to determine the time of death, it suggested that combining it with more established methods could enhance both the accuracy and reliability of time-of-death estimations.

Another key metric that can be obtained through PMCT is the cardiothoracic ratio, which assesses cardiomegaly by dividing the maximum cardiac diameter by the maximum horizontal thoracic diameter [28]. Studies have shown that elevated cardiothoracic ratios are typically found in deaths involving cardiac hypertrophy, dilation, and blood volume overload. In contrast, cases of mechanical asphyxia or drowning often exhibit a normal or slightly lower cardiothoracic ratio, as these conditions do not typically result in cardiac enlargement [29,30,31].

Recent studies on arrhythmogenic cardiomyopathy suggest that, due to the tissue replacement characteristic of this pathology, virtopsy can be particularly useful when this condition is suspected. This method also accurately distinguishes arrhythmogenic cardiomyopathy from other conditions using PMCT scans [32,33]. Moreover, the commonly used idea of comparing fist size to heart size in order to assess cardiomegaly proved to not be a reliable method, as demonstrated in a study using PMCT on 130 cases [34].

Postmortem CT (PMCT) demonstrated low sensitivity but high specificity in detecting pulmonary fat embolism as the cause of death. The study, which analyzed 830 cases, produced consistent results, with 366 cases confirmed positive through autopsy and only 18 with imaging confirmation on PMCT. Although pulmonary fat embolism is rare, it typically appears in a characteristic location within the pulmonary trunk [35]. Other modifications may be observed in the lungs through PMCT, but these do not necessarily indicate lung involvement or forensic relevance. In trauma cases, the absence of pulmonary density abnormalities associated with hypostatic phenomena should always be considered, as they are frequently linked to massive hemorrhage [36].

In cases of hepatic portal venous gas (HPVG) detected at PMCT, the autopsy also revealed bowel distension, visceral abdominal injuries, or acute circulatory dysfunction [37]. A study has also found a correlation between the severity of gastric distention and HPVG, using PMCT on 190 subjects [38].

The PMCT was successfully used in pediatric sepsis deaths due to bowel obstruction. The six pediatric cases were confirmed with septic shock, and the PMCT scans revealed bowel obstruction in all cases. There was no additional information offered by the PMMRI in these cases. Further information on existing guidelines for PMCT imaging protocols in pediatrics can be found in the work of Shelmerdine, S.C. et al. [39].

In forensics, micro-computed tomography (micro-CT) represents an advanced imaging technique, with high resolution, providing extremely detailed images of small structures. A series of cases were analyzed using micro-CT, with findings compared to postmortem histology. Results indicated that micro-CT is highly effective for fracture detection and can serve as a valuable adjunct to the histological process. However, the study did not recommend micro-CT as a substitute for histology. No cost-related aspects were discussed in this study [40,41].

The virtopsy can also be combined with other investigations. For example, after identifying and characterizing a bone fracture, a stereomicroscope can be used in charred subjects in order to analyze the exposure temperature of the bone [14]. A study suggested that biopsies guided through PMCT revealed infection-related tissue changes in 19 of 20 infective deaths [42].

Table 1 summarizes the advantages of using PMCT and the types of lesions that can be visualized using this imaging modality.

### 3.2. Angiography (PMCTA)

PMCTA has been introduced in order to assess vascular lesions in the investigation of “natural” death cases [43].

One significant challenge in PMCTA is the potential alteration of cadavers caused by the composition of the contrast medium. A study involving 20 cadavers found that an isotonic crystalloid solution with Ringer’s acetate mixed with water-soluble iodinated contrast resulted in only mild tissue alterations but exhibited moderate extravasation of the contrast medium in all cases. In contrast, a polyethylene glycol (PEG) mixture combined with water-soluble iodinated contrast, while more likely to complicate subsequent physical autopsies, was preferred for postmortem CT angiography (PMCTA) due to its superior diagnostic imaging capabilities. This preference is due to the PEG mixture’s ability to enhance contrast opacification and provide clearer visualization of vascular structures, which is crucial for accurate postmortem imaging [44].

A study emphasized the importance of selecting the appropriate contrast agent for postmortem CT angiography (PMCTA) based on the specific case. Oily contrast mixtures can alter fatty tissue within organs, potentially interfering with the assessment of conditions like liver steatosis or fatty embolism. In contrast, PEG mixtures tend to dry and fix tissues without compromising microscopic examination. Regardless of the chosen contrast medium, the study concluded that performing an autopsy promptly after PMCTA yields the most reliable results [45].

Postmortem angiography, a specialized branch of virtopsy, holds significant potential and continues to be an area of active exploration. A recent study analyzing 38 paired in vivo and postmortem CT angiography examinations focused on measuring differences in the diameters and distances of the aorta and its branches. The findings indicated higher concordance rates between in vivo and postmortem contrast-enhanced examinations, supporting the conclusion that postmortem angiography can partially replicate in vivo vascular anatomy. Furthermore, the study suggested that optimizing contrast injection techniques could further enhance diagnostic accuracy [46].

**Figure 3:**
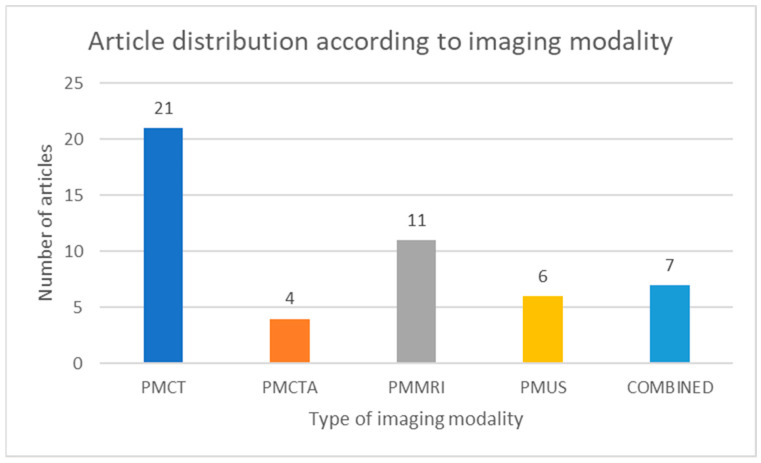
Chart depicting the distribution of the included studies considering the imaging modality used.

Figure 3 presents the distribution of the included studies according to the imaging modality used primarily.

### 3.3. Magnetic Resonance Imaging

In certain cases, PMCT provides sufficient information independently [23], making PMMRI a secondary choice with more limited applications. However, a study comparing PMCT and PMMRI in forensic investigations of brain and spinal injuries found that MRI demonstrated superior effectiveness in assessing these specific lesions [25]. Another study found that in cases of brain softening, PMMRI provided valuable diagnostic insights, particularly in assessing the junction between gray and white matter. This imaging technique also yielded valuable information on liquefied brains, whereas conventional autopsy was limited to assessing only the superior sagittal sinus in such brain conditions. The study concluded that PMMRI was the most effective examination method for this “softened” group, demonstrating superiority over both PMCT and traditional autopsy [47].

Analysis of cerebrospinal fluid using non-water-suppressed proton MRS allows for an estimation of ethanol concentration prior to autopsy; however, the measurements tend to overestimate the ethanol levels. As the method is not yet fully precise (deviations ranging from 4% to 45%), it cannot be considered a reliable source of evidence for legal proceedings [48].

The type of MRI equipment significantly influences forensic outcomes, as demonstrated by the diagnostic potential of seven Tesla MRIs, which detected minor injuries and microbleeds in patients with craniocerebral gunshot wounds, surpassing the capabilities of three Tesla MRIs. Additionally, the study highlighted that seven Tesla MRIs offer noninvasive, highly detailed soft tissue information while achieving superior spatial resolution [49].

Regarding laryngohyoid lesions, a study showed that 25% of lesions were detected only by PMMR and 17.5% only by conventional autopsy, with the results strongly suggesting that the combination of the two might capture more comprehensive results [23]. In cases of hanging, PMMRI demonstrated superior sensitivity in identifying intramuscular hemorrhages within the neck muscles compared to both autopsy and PMCT. While these hemorrhages are not strictly necessary to determine the cause of death, they offer valuable supplementary information. This can be particularly significant in ambiguous cases, where relying solely on skin marks may not sufficiently distinguish hanging from other forms of strangulation. Additionally, the ability to document such findings in greater detail could contribute to a more comprehensive forensic analysis and potentially strengthen medico-legal conclusions in contentious scenarios [50].

Both PMCT and PMMRI effectively detect postmortem cardiomyopathies; however, recent evidence suggests that in individuals under 35 years of age, PMMRI demonstrates superior accuracy [32].

During the SARS-CoV-2 pandemic, the potential brain-related contribution to respiratory distress was investigated using PMMRI. While MRI imaging revealed some hemorrhagic changes in a limited number of cases, the findings did not substantiate a direct brain-related involvement in respiratory distress. Additionally, olfactory impairments were confined to the olfactory bulbs, with no evidence of widespread neurological contributions to the respiratory symptoms [51].

PMMRI is infrequently employed in the evaluation of gunshot injuries despite its distinct advantages, including superior visualization of soft tissue damage and wound trajectories [52]. Due to the strengths of MRI in identifying wound channels and soft tissue injuries, a study investigated the potential challenges of using this imaging technique in cases involving lodged bullets, particularly the risk of metallic fragment migration [53]. PMCT examinations are significantly hindered by metal artifacts, which can obscure the bullet trajectory and conceal other potential injuries [52]. The findings demonstrated that metallic objects did not experience significant temperature increases during MRI scans, and non-ferromagnetic bullets caused minimal imaging artifacts. Additionally, ferromagnetic bullets showed a low likelihood of migration within 12 h postmortem, as they tend to align with the z-axis of the MRI’s magnetic field, resulting in orientation changes rather than physical displacement if the bullet is firmly embedded in tissue. These results highlight PMMRI as a valuable complement to PMCT in postmortem investigations of gunshot wounds [53]. Additionally, specialized CT algorithms, such as iterative metal artifact reduction, can be applied when MRI is not available [54].

To address the potential inaccuracies in postmortem interval estimation caused by post-freezing effects, a study explored the use of in situ proton magnetic resonance spectroscopy (H-MRS) on bone marrow and muscle tissue to identify prior freezing in thawed cadavers. H-MRS effectively detects whether a cadaver has been previously frozen by measuring the lip1.3/lip5 ratio and T2, lip1.3 values in bone marrow, with thresholds of a lip1.3/lip5 ratio below 20 and T2, lip1.3 under 45ms serving as reliable indicators. These changes are attributed to alterations in fatty acid chains and the reduced mobility of methylene protons, which are observable post-freezing [55].

A prospective multicenter cross-sectional study analyzed 101 cases of fetal and infant deaths that underwent both autopsy and PMMRI, along with PMCT-guided biopsy. In 91 cases, virtopsy and autopsy identified the same cause of death; however, in 45 cases, significant pathological findings were missed by virtopsy, so the conventional autopsy should still be conducted [28].

A stereomicroscopic autopsy is considered the gold standard for assessing cases of spontaneous and therapeutic pregnancy termination in first-trimester fetuses. A study involving nine cases demonstrated that seven Tesla MRIs can serve as a reliable alternative for evaluating structural anomalies, achieving a sensitivity of 94.6% and a specificity of 97.6%. One key limitation of the study was that it focused solely on structural anomalies, which may affect the overall sensitivity and specificity of the results [56]. While postmortem MRI is limited in detecting infections or hypoxic changes, it demonstrates superior capability in diagnosing conditions such as intraventricular hemorrhage, germinal matrix bleeds, and brain hematomas [57,58].

### 3.4. Postmortem Ultrasound (PMUS)

In forensics, as a postmortem radiographic modality, ultrasonography has limited use [59]. First, it is important to acknowledge that refrigeration and the hardening of adipose tissue can significantly influence the penetration and reflection of ultrasound waves. Furthermore, due to the presence of decomposition gases, it is recommended that ultrasound be performed within 4 to 5 days postmortem to ensure more reliable results [60]. The presence of rigor mortis and the associated stiffness of the deceased can significantly limit the lateral acoustic window, complicating ultrasound assessment of deeper structures such as the liver, spleen, kidneys, lung bases, or aorta, particularly after rigor mortis has fully developed [59].

A comprehensive study by Uchigasaki S. et al. explored the applications of postmortem ultrasound (US) in forensic pathology. These applications include cardiovascular assessments, such as detecting abdominal aortic aneurysms, pericardial tamponade, and cardiac hypertrophy. Additionally, postmortem US is effective for examining various organs, including the liver, pancreas, kidneys, and spleen, and identifying pleural effusions or infectious conditions such as subphrenic abscesses [61]. A portable ultrasound device was utilized on corpses at varying stages of decomposition, successfully identifying numerous pathological findings, which were subsequently confirmed through autopsy. The primary advantage of this approach lies in its portability, allowing the device to be brought directly to the forensic setting, thereby facilitating faster preliminary assessments [62].

In the absence of other virtopsy imaging modalities, ultrasound imaging represents a safe alternative examination [60] in the cases mentioned above. Lung ultrasound, with its high diagnostic accuracy of 90.5% in cases of acute respiratory failure among critically ill patients, was employed during the COVID-19 pandemic on deceased individuals to minimize the risk of infection transmission to healthcare staff while enabling postmortem assessments [63].

In 2002, a novel technique known as ultrasonographic autopsy, or echopsy, was developed to address the challenges of obtaining biopsy material through needle autopsy. This method involved sample collection under sonographic guidance and was demonstrated to be a feasible and reliable alternative to traditional autopsy procedures when the traditional autopsy is not feasible [64], although it cannot substitute conventional autopsy [65]. US-guided tissue aspiration has also been tested and provided promising results [64].

A pure US-guided approach was conducted on 10 dead bodies in order to demonstrate the catheter’s correct positioning using this technique in 94% of cases. The catheter location was verified using PMCT. The importance of postmortem US, in this case, was to demonstrate that this technique significantly reduced misplacements outside the paravertebral space [66].

In cases of fetal demise, the US can provide valuable information on the brain, lungs, and abdomen due to the absence of inhaled air [67].

## 4. Discussions

Even though constant effort is put into post-autopsy reconstructions through various new techniques, emerging technologies can also be encompassed within the broader framework of virtopsy. Among these is mobile energy-dispersive X-ray fluorescence (EDXRF), which emphasizes chemical analysis over anatomical imaging. Its utility is particularly notable in dental forensics, where it has demonstrated the capability to distinguish between 36 different types of dental filling materials [68].

As anticipated, both PMCT and PMMRI produce high-resolution images with advanced, detailed reconstructions, offering substantial utility in postmortem examinations. A recent study investigating causes of sudden death alongside virtopsy findings highlighted the particular value of PMCT in examining adults, whereas PMMRI was found to be more beneficial in younger decedents [69]. Since virtopsy has demonstrated significant value in forensic medicine, new techniques have been developed to achieve superior spatial and contrast resolution, enhancing imaging capabilities for more accurate material differentiation. One study, for instance, explored the use of photon-counting CT in forensic death investigations, revealing several potential advantages associated with this method [70].

There is a growing interest in leveraging artificial intelligence (AI) to streamline workflows across various disciplines. Advanced neural networks and deep learning algorithms facilitate the precise customization of AI models to suit specific tasks, enhancing efficiency and accuracy in decision-making processes [71]. Forensic researchers have conducted studies based on AI in recent years, aiding them in tasks such as face recognition, age and gender identification, cause of death identification, DNA analysis, enhancing estimation of ballistics, and even aiding forensic toxicology [72,73]. AI is considered a technology that will revolutionize the virtopsy, as a review study explored its applicability in various forensic articles. Most of them enhanced the identification analysis of radiological data, enhancing the estimation of ballistics and even aiding forensic toxicology [72]. Using maximum and minimum intensity projections, a study utilized PMCT images to successfully develop an algorithm capable of automating the visualization of gas distribution in the thorax and abdomen, detecting radiopaque foreign bodies, and mapping skeletal structures [74]. Despite the advanced resolution of seven Tesla MRI scanners, they are still unable to detect microstructural changes critical to studying neurodegenerative diseases. A comparison of postmortem MRI images with histological findings reveals the limitations of current imaging techniques, underscoring the need for artificial intelligence to accelerate research on neurodegenerative progression. The integration of AI algorithms could bridge the gap between imaging and neuropathology, ultimately facilitating the development of strategies for disease prevention and treatment [47]. Indeed, a balance must be maintained between the use of AI and ethical guidelines to ensure the preservation of trust and integrity in forensic practices [72].

Our findings align with previous studies demonstrating a high level of concordance between virtopsy and conventional autopsy in determining the cause of death. However, they also reveal that virtopsy exhibits lower accuracy in evaluating major pathologies [41]. While virtopsy is effective in identifying lethal injuries, particularly in cases of traumatic or unnatural deaths, ref. [75] it may fail to detect subtle or complex findings, such as microscopic tissue changes, chemical traces, or histopathological alterations. These limitations highlight the need for complementary methods for comprehensive forensic evaluation.

The proposed imaging techniques still require standardization in order to become stronger than common autopsy in order to be considered faithful evidence in forensics [18].

**Figure 4:**
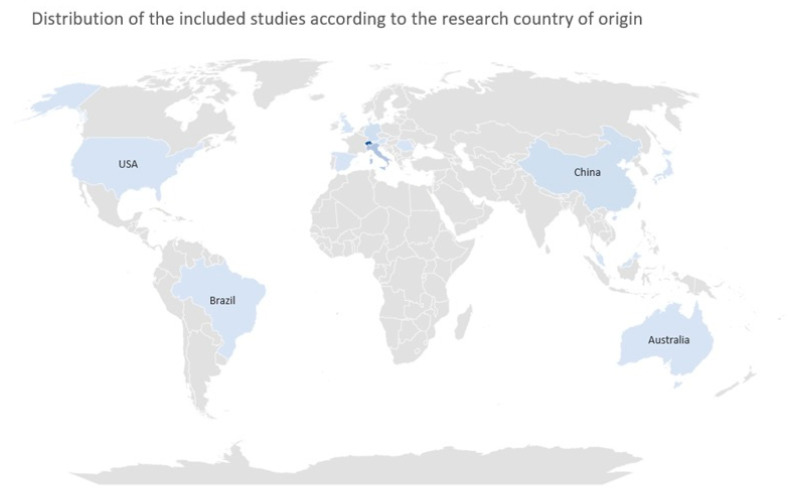
Chart presenting the geographical distribution of the articles included in the present scoping review.

Interestingly, the analysis of the geographical origin of the articles included in this scoping review revealed a strong interest in the development of virtopsy in the Asian and European continents, as shown in Figure 4.

Table 2 comprises the advantages and disadvantages of virtopsy.

While virtopsy excels in non-invasive evaluations, its limitation in providing detailed tissue-level insights is hindered by the quality of images and resolution. Histological samples remain the gold standard for tissue evaluation and, furthermore, can be helpful in post-mortem interval detection [76].

The limitations of this study stem from the heterogeneity of the data regarding causes of death, which challenges the uniformity and generalizability of the findings. Additionally, including case reports while providing detailed insights into specific instances does not contribute significantly to statistical robustness or broader applicability. Furthermore, no blind studies were included, limiting the ability to control for potential observer bias and reducing the overall strength of the evidence presented.

## 5. Conclusions

In situations where a traditional autopsy is either prohibited or unfeasible, a virtopsy can provide valuable insights into the cause of death. However, there is no evidence suggesting that virtopsy should replace traditional autopsy, as each virtopsy technique has inherent limitations. Current recommendations emphasize combining various virtopsy methods to improve diagnostic accuracy and enhance forensic investigations.

PMCT is highly effective for detecting complex osseous injuries, tracing bullet trajectories, identifying characteristic findings in drowning cases, examining intervertebral disks, and spotting Simon’s bleeding. It is also useful for assessing the cardiothoracic ratio and distinguishing arrhythmogenic cardiomyopathy from other conditions. While PMCT shows high specificity in detecting pulmonary fat embolism, its sensitivity remains low.

PMCTA is valuable for evaluating vascular lesions, particularly in natural death cases. However, the use of contrast medium can alter cadaveric tissue and impact autopsy quality.

PMMRI is superior in analyzing soft tissues, including brain and spinal structures, cerebrospinal fluid, microbleeds, and laryngohyoid lesions. It is especially useful for identifying cardiomyopathies in individuals under 35 years old and assessing soft tissue damage in gunshot cases.

PMUS serves as an alternative when PMCT and PMMRI are unavailable. Its portability also allows for use in forensic settings.

## Figures and Tables

**Table 1:** Use of PMCT.

Use of PMCT	Reference
Complex spinal fractures	[11]
Severe polytrauma	[12]
3D reconstruction of facial injuries	[13]
Trace bullet trajectory	[18]
Hanging deaths	[21]
Cases of drowning	[22]
Intracranial gas accumulation	[25]
Cardiothoracic ratio measurement	[31]
Arrhythmogenic cardiomyopathy	[33]
Pulmonary fat embolism	[35]
Guided biopsy	[42]

**Table 2:** Advantages and disadvantages of virtopsy.

Advantages	Disadvantages
Body preservation	Needs to differentiate pathology from decomposition.
Solves cultural beliefs	Cannot differentiate between the entry and exit points of the projectile.
Limits the contamination of the environment with radiation, toxins, or infectious agents	Skin changes are not visible.
Mobile imaging units	Can imply supplementary equipment costs.
Records available for the examination of the judiciary system	Imaging specialists need to be trained in the analysis of body decomposition.

## Data Availability

All data are available from the corresponding author upon reasonable request due to the large size of the dataset.
